# 1D-Zigzag Eu^3+^/Tb^3+^ Coordination Chains as Luminescent Ratiometric Thermometers Endowed with Multicolor Emission

**DOI:** 10.3390/ma14216445

**Published:** 2021-10-27

**Authors:** Luca Bellucci, Gregorio Bottaro, Luca Labella, Fabio Marchetti, Simona Samaritani, Daniela Belli Dell'Amico, Lidia Armelao

**Affiliations:** 1 Istituto di Chimica della Materia Condensata e di Tecnologie per l'Energia, Consiglio Nazionale delle Ricerche, ICMATE-CNR and INSTM, Dipartimento di Scienze Chimiche, Università di Padova, via Marzolo 1, I-35131 Padova, Italy; luca.bellucci@phd.unipd.it; 2 Dipartimento di Chimica e Chimica Industriale and CIRCC, Università di Pisa, via Giuseppe Moruzzi 13, I-56124 Pisa, Italy; fabio.marchetti@unipi.it (F.M.); simona.samaritani@unipi.it (S.S.); daniela.belli@unipi.it (D.B.D.); 3 Dipartimento di Scienze Chimiche and INSTM, Università di Padova, via Marzolo 1, I-35131 Padova, Italy; lidia.armelao@unipd.it; 4 Dipartimento di Scienze Chimiche e Tecnologie dei Materiali (DSCTM), Consiglio Nazionale delle Ricerche, Piazzale A. Moro 7, 00185 Rome, Italy

**Keywords:** luminescence, europium, terbium, lanthanide coordination polymers, luminescent molecular thermometers, ratiometric thermometers

## Abstract

Two homometallic Coordination Polymers (CPs) with composition [Ln(hfac)_3_bipy]_n_ (Ln^3+^ = Eu^3+^, **1**, and Tb^3+^, **2**; hfac = hexafluoroacetylacetonato, bipy = 4,4′-bipyridine) were used to develop a family of ratiometric luminescent thermometers containing Eu^3+^ and Tb^3+^ as red and green emitters, respectively. The thermometric properties of pure CPs and of their mixtures having an Eu^3+^/Tb^3+^ molar ratio of 1:1, 1:3, 1:5, and 1:10 (samples: **Eu1Tb1**, **Eu1Tb3**, **Eu1Tb5**, and **Eu1Tb10**) were studied in the 83–383 K temperature range. Irrespective of the chemical composition, we observed similar thermometric responses characterized by broad applicative temperature ranges (from 100 to 165 K wide), and high relative thermal sensitivity values (S_r_), up to 2.40% K^−1^, in the physiological temperature range (298–318 K). All samples showed emissions endowed with peculiar and continuous color variation from green (83 K) to red (383 K) that can be exploited to develop a colorimetric temperature indicator. At fixed temperature, the color of the emitted light can be tuned by varying composition and excitation wavelength.

## 1. Introduction

Nowadays, luminescence thermometry plays a relevant role due to its high importance in many societal needs. For example, contactless temperature measurements with a sub-micrometric spatial resolution are required in countless technological applications and industrial fields, such as microelectronics, microoptics, photonics, microfluidics, and nanomedicine (e.g., in intracellular temperature measurements, or in microcircuit thermal map) [1,2,3]. Among the different types of luminescent thermometers, intensity-based ones are particularly useful for many applications such as real-time measurements on large moving systems [4,5]. However, using a single emitter, fluctuations of the light source or changes in the emitter local concentration are potential sources of error in temperature readout [6,7,8].

The stated drawbacks can be easily overcome using the intensity ratio of different emission bands of a given luminescent material, thus creating self-referencing thermometers [6,7,9,10]. These thermometers, also called “ratiometric thermometers”, have been developed using a variety of luminescent probes [7,8,11]. Among others, europium and terbium complexes have been extensively used due to their peculiar luminescent properties [6,12,13,14,15,16,17].

Lanthanide Metal–Organic Frameworks (MOFs) and Coordination Polymers (CPs) immediately appeared to be excellent platforms for the development of ratiometric thermometers [3,11,15,17,18]. Indeed, the possibility to introduce different metal ions simultaneously in MOFs and CPs, without altering their structure, marked them as two of the most promising materials for the development of luminescent thermometers. Furthermore, the rational fine tuning of their luminescent properties simply varying the nature of the different building blocks (e.g., metal ions, spacer ligands, and guest molecules) or their sequence within the network, i.e., the so-called “spatial composition*”* [19,20], give countless possibilities for the creation of systems with desired properties [11,21,22]. Nevertheless, dual metal center thermometers allow to associate temperature to the color of emitted light, making them particularly appealing for the development of colorimetric temperature sensors [23,24].

Polycarboxylates ligands have been extensively used for the development of luminescent thermometers based on Eu^3+^/Tb^3+^ MOFs and CPs [3,11,13,17,25,26] due to the possibility of choosing from a wide variety of commercial/easily synthesized divergent ligands whose photoluminescent properties were suitable for the design of luminescent compounds. Conversely, examples of lanthanides MOFs and CPs endowed with thermometric properties and based on β-diketonate (β-dike) ligands are relatively scarce in the literature [27,28], though β-dike ligands have been widely employed in the synthesis of low-nuclearity lanthanide luminescent complexes with thermometric properties [9,29,30,31,32].

Recently, we developed a convenient strategy for the synthesis of a series of highly luminescent one-dimensional lanthanide zigzag polymeric chains with composition [Ln(β-dike)_3_(bipy)]_n_ based on lanthanide *tris*-β-diketonato complexes as nodes (for Ln^3+^ = Eu^3+^ and Tb^3+^; β-dike = dibenzoylmethide, dbm; while for Ln^3+^ = Eu^3+^ β-dike = thenoyltrifluoroacetonate, tta, or hexafluoroacetilacetonate, hfac) and 4,4′-bipyridine (bipy) as neutral spacer [33,34]. To develop a heterometallic ratiometric thermometer, the “*antenna ligand*”, i.e., the organic molecule that sensitizes lanthanide emission [35,36], must have a triplet energy level high enough to promote the indirect excitation of both lanthanides ions. According to this requirement, the β-diketonate ligand hexafluoroacetylacetonate is a good choice for the development of europium and terbium-based ratiometric luminescent thermometers, since its excited triplet state (*T* ≈ 21,900 cm^−1^) [37] has energy higher than europium ^5^D_0_ (≈17,200 cm^−1^) [38] and terbium ^5^D_4_ (≈20,500 cm^−1^) [31] emissive levels.

In this work, we studied a family of self-calibrating ratiometric thermometers, consisting of [Eu(hfac)_3_(bipy)]_n_ and [Tb(hfac)_3_(bipy)]_n_ homometallic CPs and their mixture in a KBr matrix, endowed with temperature- and composition-dependent emission intensity and color between 83 and 383 K. We preferred to mix the two homometallic CPs in an inert matrix over the direct synthesis of the corresponding heterobimetallic CPs to properly control spatial composition [19,20] of the samples. In fact, due to the similar chemical properties [39,40] of Eu^3+^ and Tb^3+^, the synthesis of heterobimetallic CPs will result in zigzag polymeric chains having random, statistically defined, —(M—M’)_n_—sequences (— = bridging ligand, M (M’) = Eu^3+^ and/or Tb^3+^).

Irrespective of the composition, the obtained materials are characterized by good thermometric properties in the 213–383 K temperature range. We highlighted how the color variation of the emitted light is exploitable to develop colorimetric thermometers even using the eyes as detector. This feature is of particular interest because it evidences the possibility for the spectroscopy-to-imaging readout transition for luminescence thermometers that would greatly increase their use and implementation into more complex tags, like QR-codes, easily readable with smartphones [24].

## 2. Materials and Methods

Anhydrous toluene was purchased from Merck and used without further purification. [Eu(hfac)_3_] and [Tb(hfac)_3_] were prepared from the corresponding bi-hydrated complex [Ln(hfac)_3_(H_2_O)_2_] according to the literature [33]. 4,4′-bipyridine (bipy, Aldrich) was used as received. All reactions have been carried out under inert Ar atmosphere using standard Schlenk techniques.

FTIR spectra in solid phase were recorded with a Perkin–Elmer ‘‘Spectrum One’’ spectrometer, equipped with an ATR accessory (Perkin Elmer, Waltham, MA USA). Elemental analyses (C, H, N) were performed at the Dipartimento di Chimica e Chimica Industriale, Università di Pisa (Pisa, Italy). The metal content was determined by treating the samples with diluted HNO_3_ in a platinum crucible. The resulting solution was then evaporated to dryness. After calcination at 850 °C, solid residues were weighted as Eu_2_O_3_ or Tb_2_O_3_.

Photoluminescence experiments have been performed on KBr pellets (Sigma-Aldrich, spectroscopic grade, Sigma-Aldrich, Burlington, MA, USA) containing pure CPs or their mixtures. KBr has been previously heated at 100 °C overnight to remove H_2_O traces. Room temperature luminescence spectra were recorded in a front-face acquisition geometry with a Horiba JobinYvon *Fluorolog-3* spectrofluorimeter (Horiba JobinYvon, Edison, NJ, USA) equipped with double-grating monochromator in both the excitation and emission sides, coupled to a *R928P* Hamamatsu photomultiplier and a 450 W Xe arc lamp as the excitation source. Emission spectra were corrected for detection and optical spectral response of the spectrofluorimeter supplied by the manufacturer. The excitation spectra were corrected for the spectral distribution of the lamp intensity using a photodiode reference detector.

Temperature-dependent experiments (83–383 K) were carried out in backscattering geometry using a Horiba T64000 triple spectrometer equipped with a Peltier-cooled charge-coupled device detector (Horiba Synapse). A Xe arc lamp (450 W) has been used as excitation source. The scattered radiation was collected through a 10× microscope objective (Olympus MPLAN, 10 × 0.25, Olympus, Tokyo, Japan). The spectrograph, equipped with 300 lines/mm gratings, was used as a single-stage imaging monochromator. Temperature-dependent experiments were performed by means of a Linkam THMS600 heating/freezing microscope (Linkam, Tadworth, UK) stage having temperature stability <0.1 K over the 83–873 K temperature range.

### 2.1. Synthesis of [Eu(hfac)_3_(bipy)]_n_ (1)

[Eu(hfac)_3_] (0.603 g; 0.78 mmol) was suspended in toluene (90 mL), and 4,4′-bipyridine (0.125 g; 0.80 mmol) was added. After 2 h stirring at about 60 °C, the yellowish solution was slowly cooled to room temperature. The precipitated solid was filtered off and dried in vacuo at room temperature for four hours (0.543 g yield 75.0% as [Eu(hfac)_3_(bipy)]). El. Anal. Calc. for [Eu(hfac)_3_(bipy)], C_25_H_11_EuF_18_O_6_N_2_: C, 32.3; H, 1.2; Eu, 16.4; N, 3.0. Found: C, 32.6; H, 1.3; Eu, 16.6; N, 2.8%. ATR IR: (range: 1700–700 cm^–1^) 1647 (s, νC=O), 1608 (mw, νC=C), 1558 (mw, νC=O + δC-H), 1534 (m), 1461 (ms), 1418 (mw), 1251 (s), 1199 (s), 1141 (s), 1101 (s, νC-F), 1069 (m), 1047 (mw), 1005 (mw), 972 (w), 951 (w), 858 (w), 800 (ms), 767 (w), 733 (m).

### 2.2. Synthesis of [Tb(hfac)_3_(bipy)]_n_ (2)

[Tb(hfac)_3_] (0.998 g; 1.28 mmol) was suspended in toluene (100 mL) and 4,4′-bipyridine (0.200 g; 1.28 mmol) was added. The suspension was heated for 2 h at 60 °C, obtaining a yellow solution which was slowly cooled to room temperature. A suspension of a microcrystalline solid was obtained, filtered, and dried in vacuo at room temperature for four hours (1.019 g yield 85.0% as [Tb(hfac)_3_(bipy)]). El. Anal. Calc. for [Tb(hfac)_3_(bipy)], C_25_H_11_TbF_18_O_6_N_2_: C, 32.1; H, 1.2; N, 3.0; Tb, 17.0. Found: C, 32.0; H, 1.2; N, 3.3%; Tb, 17.8. ATR IR: (range: 1700–700 cm^–1^) 1647 (s, νC=O), 1608 (mw, νC=C), 1558 (mw, νC=O + δC-H), 1534 (m), 1461 (ms), 1418 (mw), 1251 (s), 1199 (s), 1141 (s), 1101 (s, νC-F), 1069 (m), 1047 (mw), 1005 (mw), 972 (w), 951 (w), 858 (w), 800 (ms), 767 (w), 733 (m).

### 2.3. Preparation of KBr Pellets Containing [Ln(hfac)_3_(bipy)]_n_ (Ln^3+^ = Eu^3+^, Tb^3+^) CPs

Known amounts of [Ln(hfac)_3_(bipy)]_n_ (Ln^3+^ = Eu^3+^, Tb^3+^) were dispersed in the proper amount of dried KBr to obtain the desired europium/terbium molar ratio. The total lanthanide (Eu^3+^ + Tb^3+^) molar concentration was kept constant. The mixtures were grinded in an agate mortar, adding few drops of n-pentane, dried under a gentle N_2_ flow, and then pressed, forming the pellets. Six pellets were prepared, two containing the pure CPs (1 and 2) and four made of a mixture of the two homometallic CPs with a 1:1, 1:3, 1:5, or 1:10 Eu^3+^/Tb^3+^ molar ratio (**Eu1Tb1**, **Eu1Tb3**, **Eu1Tb5**, and **Eu1Tb10**). Materials composition is summarized in Table 1**.** The reproducibility in samples preparation was investigated through photoluminescence experiments comparing the intensity ratio between Eu^3+ 5^D_0_→^7^F_2_ (614 nm) and Tb^3+ 5^D_4_→^7^F_5_ (543 nm) transitions (I_Eu_/I_Tb_) on three different replicates of **Eu1Tb3**. An experimental error of ± 10% was found.

## 3. Results and Discussion

### 3.1. Synthesis and Characterization

**Figure 1:**
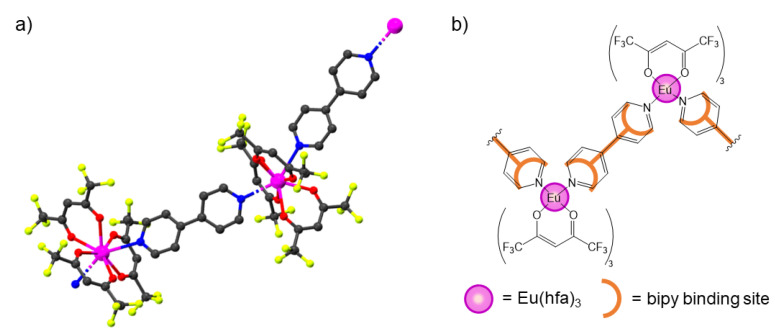
(**a**) Structure of a portion of the chain of [Eu(hfac)_3_(bipy)∙toluene]_n_. Toluene molecules were omitted for clarity. CCDC number 1471967, from ref. [33]. (**b**) Schematic representation of [Eu(hfac)_3_(bipy)∙toluene]_n_.

In our previous work, we carefully described the synthesis of monodimensional lanthanide β-diketonate coordination polymers based on bipy as connector ligand [33]. The synthesis of these CPs must be carried out in anhydrous toluene, avoiding the use of oxygenated solvents. Indeed, the presence of O-donor molecules in the reaction environment led to the formation of low-nuclearity products due to the higher affinity of lanthanide ions towards oxygenated ligands than to N-donor ones [41,42,43]. We also showed that 1D zigzag CPs were obtained regardless of the employed [Ln(β-dike)_3_] precursors, using an equimolar amount of bipy per metal center [33]. We also reported the synthesis and the characterization of [Eu(hfac)_3_(bipy)]_n_ (**1**). Its structure consists of 1D zigzag chains extended along the b crystallographic axis (Figure 1). Each europium center is coordinated to six oxygen atoms from the three hfac ligands and two N atoms of two different bipy in a distorted square antiprismatic geometry [33].

The terbium CP [Tb(hfac)_3_(bipy)]_n_ (**2**) has been similarly synthesized, reacting [Tb(hfac)_3_] and bipy in a 1:1 molar ratio, and characterized through elemental analysis and infrared spectroscopy (Figure S1).

### 3.2. Photoluminescence and Thermometric Studies

**Figure 2:**
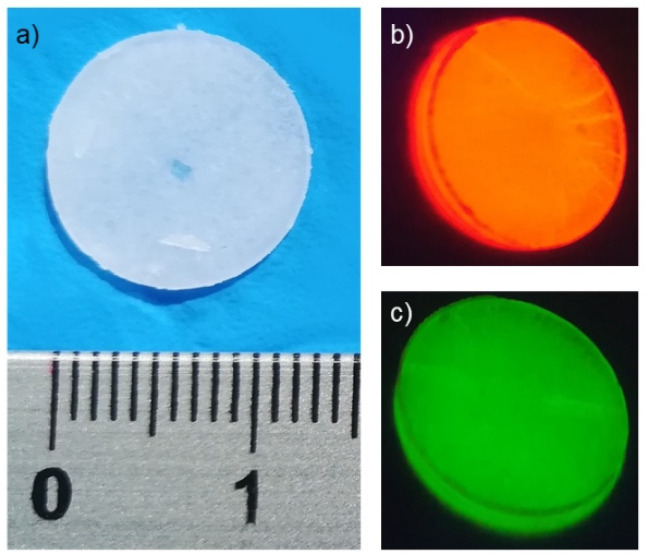
(**a**) Picture of a pellet illuminated with white light (the length scale is in cm); and (**b**,**c**) picture of KBr pellets containing **1** (**b**) and **2** (**c**) exposed to UV light (λ_exc_ =320 nm) at room temperature.

Upon irradiation, **1** and **2** emissions are excited over a wide wavelength range, from UV to visible, up to ≈450 nm for **1** and ≈400 nm for **2** (Figure S2). The lanthanides-sensitized emission can be achieved through the so-called “antenna effect” exploiting the absorption properties of the organic ligands. The process can be summarized in the three following steps: (i) absorption of light by the organic ligand and population of its first excited singlet state (S_1_); (ii) intersystem crossing (ISC) from the singlet to the triplet (T_1_) level, and (iii) energy transfer (E.T.) from T_1_ to the lanthanide excited level [44]. Both the compounds showed intense light emission, also visible at the naked eye (Figure 2), red for **1** and green for **2**. 

**Figure 3:**
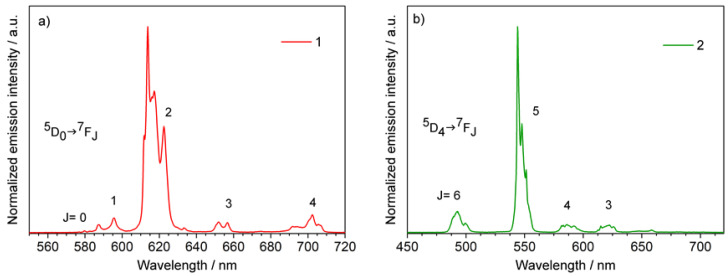
Emission spectra of compounds (**a**) **1** and (**b**) **2**. λ_exc_= 320 nm.

In particular, the emission spectrum of **1** (Figure 3a) is characterized by the typical Eu^3+ 5^D_0_→^7^F_J_ (J = 0–4) transitions corresponding to the bands centered at 587, 595, 614, 652, and 693 nm. Conversely, compound **2** emission spectrum (Figure 3b) showed Tb^3+ 5^D_4_→^7^F_J_ (J = 6–3) transitions centered at 490, 543, 583, and 619 nm, respectively [45]. The spectra (Figure 3) of **1** and **2**, dominated by the ^5^D_0_→^7^F_2_ (Eu^3+^) and ^5^D_4_→^7^F_5_ (Tb^3+^), have a shape that is typical for β-diketonato complexes [38,46].

The photoluminescence excitation (PLE, see Figure S2) spectrum of **2** is predominantly localized at λ < 380 nm with a low-intensity tail extending to the boundary with the visible region. Instead, the PLE spectrum of **1** is redshifted and extends in the visible up to about 450 nm. Based on the shape of PLE spectra, we decided to study **1**, **2** and their mixtures, exciting their luminescence at 320 and 370 nm. While at 320 nm both emitters are effectively excited, PLE signals of **1** at 370 nm are more intense than for **2.** However, the use of 370 nm excitation wavelength has distinctive applicative and technological advantages compared to 320 nm, being compatible with organic substrates used in flexible electronics and with low glass transition temperature glasses having a cutoff wavelength around 350 nm. Moreover, light-emitting devices (LEDs) emitting at 370 nm are quite common, and generally cheaper than UV LED.

**Figure 4:**
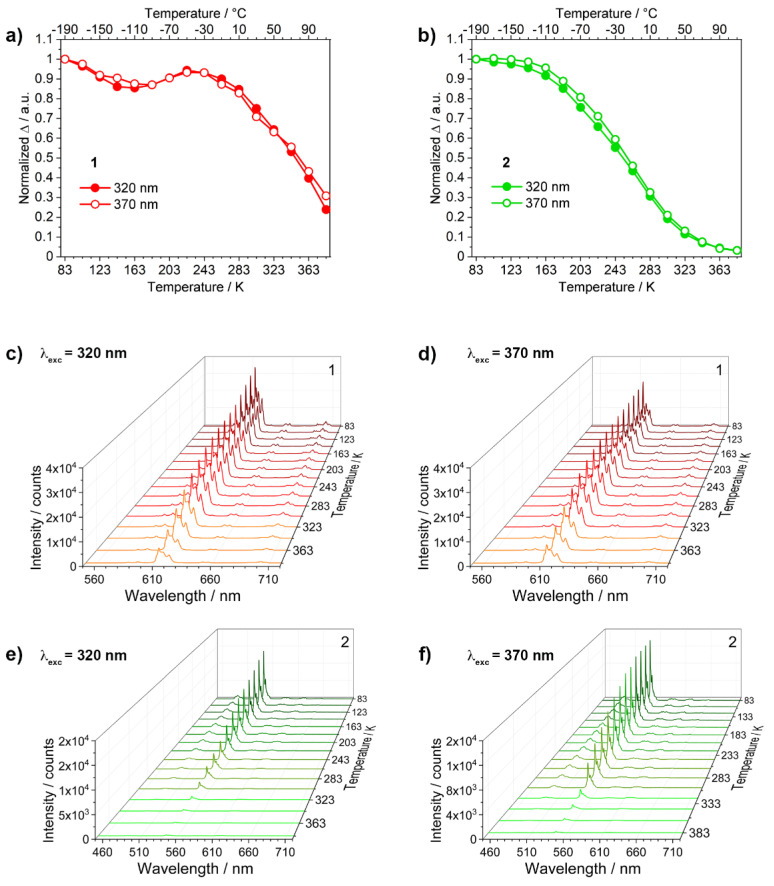
Comparison between the Δ curves of compounds (**a**) **1** and (**b**) **2**. λ_exc_= 320 nm (●) and 370 nm (○). Emission spectra of **1** (**c**) λ_exc_= 320 nm, (**d**) λ_exc_= 370 nm, and **2** (**e**) λ_exc_= 320 nm, (**f**) λ_exc_= 370 nm.

The temperature-dependent luminescence properties of compounds **1** and **2** were studied in the 83–383 K temperature range. As visible from Figure 4c–e, the emission intensity of both compounds decreases as temperature increases. The integrated areas of Eu^3+ 5^D_0_→^7^F_2_ and Tb^3+ 5^D_4_→^7^F_5_ transitions were chosen as thermometric parameter (Δ) for **1** and **2**, respectively, since they are the main contributions in the total emission spectra. The Δ curves of **1** and **2**, normalized at 83 K, are reported in Figure 4.

The shape of Δ curves depends on the employed metal ion. In **1** (Figure 4a), the thermometric parameter has small variations up to ≈223 K, and then decreases, keeping 25% ca. of the initial ^5^D_0_→^7^F_2_ transition intensity at 383 K (Figure 4c,d). This trend does not depend on the employed excitation wavelength. Conversely, compound **2** showed a well-defined S-shaped profile that gradually decreases above ≈113 K. Under the adopted experimental conditions, **2** did not show residual emission intensity at 383 K (Figure 4b,e,f). The emission of terbium-CP is partially quenched at room temperature, although still clearly observable, and gains intensity lowering the temperature. Europium emission is less effected and is very bright from physiological down to cryogenic temperatures (T < 100 K) [27,39]. The shape of Δ vs. T curves depends on the balance between radiative and non-radiative deactivation pathways that contribute to the modulation of the luminescence properties. The most encountered non-radiative processes determining the overall thermometric response of the luminescent molecular thermometers are: (i) back energy transfer from Ln^3+^ to the ligand triplet level, (ii) multiphonon relaxation promoted by high energy oscillators (mainly −OH and −NH groups) directly bonded to the metal center, and (iii) energy transfer to LMCT states. Depending on the activation energies, more than one process can operate. Other deactivation paths can also be encountered but are less common (ion–ion interaction, donor-acceptor phenomena, etc.). The different behavior displayed by **1** and **2** is mainly correlated to the energy difference between Eu^3+^ or Tb^3+^ emitting levels and antenna triplet level (ca. 4700 and 1500 cm^−1^ for Eu^3+^ and Tb^3+^, respectively) that affect the efficiency of the energy transfer from T_1_ to the lanthanide excited level, i.e., from the back energy transfer that results more efficient in **2**. A contribution from LMCT states cannot be unambiguously ruled out [44].

For a better comparison of compounds **1** and **2** thermometric properties, we used the relative thermal sensitivity (S_r_, % K^−1^) [6], a figure of merit commonly adopted to compare thermometers was used:

(1) $$S_{r}=\frac{1}{\Delta }\left|\frac{\delta \Delta }{\delta T}\right|$$

In Equation (1), Δ is the thermometric parameter and δΔ is the variation of the thermometric parameter on a certain temperature variation (δT). A value of S_r_ ≥ 1 was assumed as quality criterion to determine the applicative temperature range of these CPs [3].

**Figure 5:**
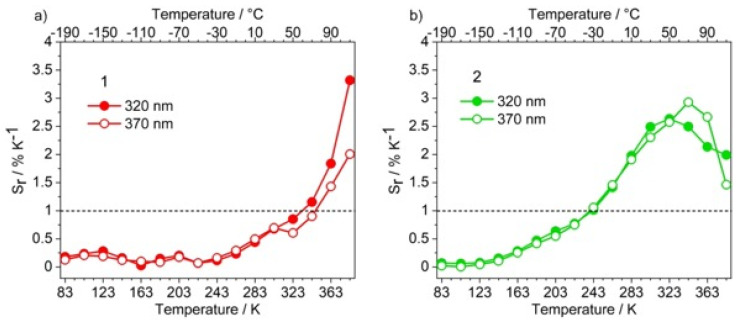
Comparison between the S_r_ curves of compounds (**a**) **1** and (**b**) **2.** λ_exc_= 320 nm (●) and 370 nm (○).

Below 263 K, compound **1** (Figure 5a) has S_r_ close to zero. Instead, S_r_ is >1 above 323 K and its temperature-dependence is almost independent from the excitation wavelengths. Conversely, **2** (Figure 5b) has S_r_ ≠ 0 in almost all the studied temperature range, with an application range from 243 to 383 K. 

### 3.3. Development of Eu^3+^-Tb^3+^ Ratiometric Thermometers

**1** and **2** are sensitive to temperature variation and can be efficiently employed to develop a family of ratiometric luminescent thermometers using the ^5^D_4_→^7^F_5_ (Tb^3+^)/^5^D_0_→^7^F_2_ (Eu^3+^) intensity ratio as thermometric parameter (Δ = I_Tb_/I_Eu_).

Known amounts of the two homometallic CPs (see Table 1) were mixed in KBr, obtaining homogeneous dispersions. The mixtures have been finely grinded in an agate mortar, adding a few drops of n-pentane to facilitate the milling process. The organic solvent was then removed under N_2_ flow. Finally, the resulting powders have been pressed, forming non-friable easy-to-handle pellets. Four samples with different europium/terbium molar ratios have been prepared, keeping constant the Ln^3+^/KBr molar ratio (Ln^3+^ = Eu^3+^ + Tb^3+^). The samples were labelled **Eu1Tb1**, **Eu1Tb3**, **Eu1Tb5**, and **Eu1Tb10** according to the Eu^3+^/Tb^3+^ relative amount (1:1, 1:3, 1:5, and 1:10 respectively).

**Figure 6:**
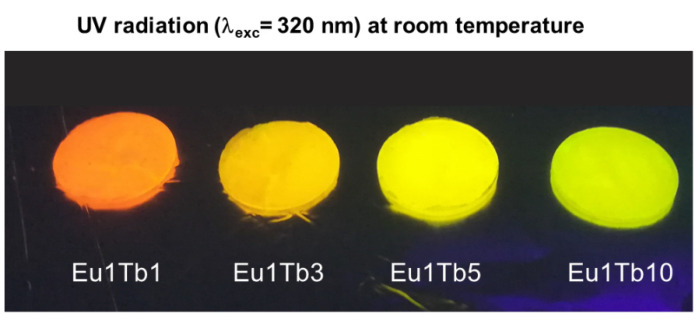
Room temperature Eu-Tb mixed samples appearance under UV irradiation. λ_exc_= 320 nm.

**Figure 7:**
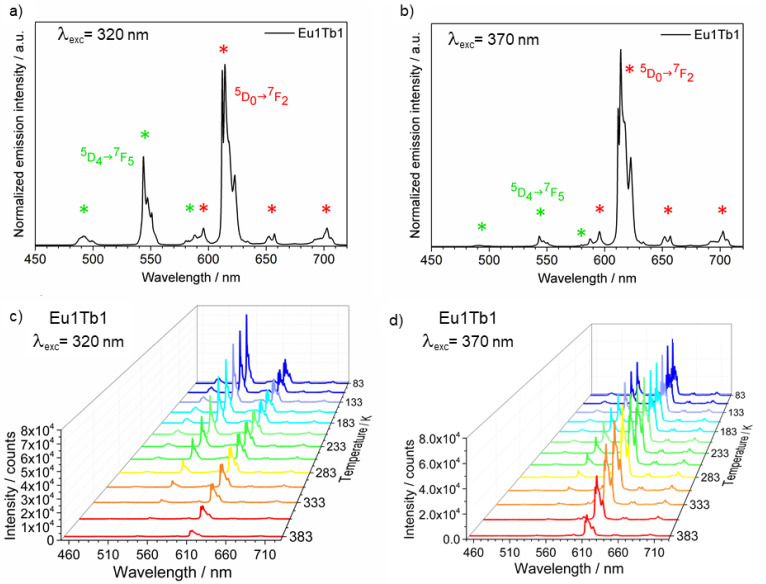
(**a**,**b**) Room temperature and (**c**,**d**) temperature-dependent emission spectra of sample **Eu1Tb1**. (**a**–**c**) λ_exc_ = 320 nm, (**b**–**d**) λ_exc_= 370 nm. In (**a**,**b**) Tb^3+^emissions are labelled with green asterisks, while Eu^3+^ transitions with red ones.

At room temperature, the samples showed a bright homogeneous emission also visible to the naked eye. The color of the emitted light is a combination of the Eu^3+^ (red) and Tb^3+^ (green) emissions, which varies from orange to pale green, increasing the terbium content in the mixture (see Figure 6, Figure 7a,b, Figures S3 and S4).

Switching the excitation wavelength from 320 nm to 370 nm, we modified the relative intensity of Eu^3+^ and Tb^3+^ emissions (compare Figure 7a,b, Figures S3 and S4), since the longer wavelengths favor europium emission with respect to the terbium one (see Figure S2). To check the sample homogeneity, we measured the emission spectra (λ_exc_= 320 nm and 370 nm) on different spots on each pellet (sampling size = 200 µm). We observed small differences in the I_Tb_/I_Eu_ intensity ratio, within 10%, thus confirming a homogeneous dispersion of the two CPs inside the specimens.

The temperature-dependent luminescent properties of the mixed samples were studied in the 83–383 K range. As an example, emission spectra of **Eu1Tb1** at 320 nm and 370 nm recorded at different temperatures are reported in Figure 7c,d (see also Figures S5 and S6 for the other samples).

Noteworthy, in all the samples the intensity of terbium ^5^D_4_→^7^F_5_ transition is strongly influenced by temperature variations, while the europium ^5^D_0_→^7^F_2_ band is less affected (Figure 7c,d, Figures S5 and S6), as also previously observed in the homometallic CPs **1** and **2**. The color of the emitted light changes from green/yellow to orange/red depending on the sample composition and the excitation wavelength going from low to high temperature.

**Figure 8:**
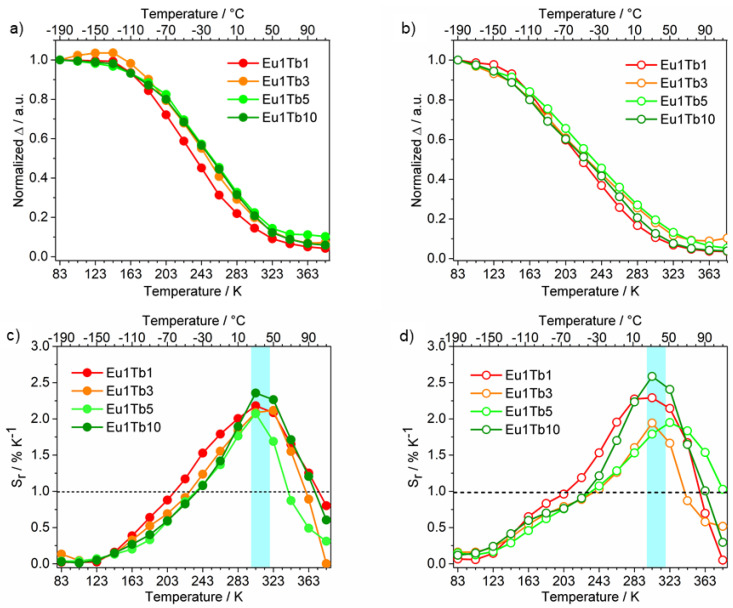
(**a**,**b**) Normalized Δ vs. T and (**c**,**d**) S_r_ curves of Eu−Tb mixed samples; in light blue is reported the physiological temperature range. (**a**–**c**) λ_exc_ = 320 nm, (**b**–**d**) λ_exc_ = 370 nm.

For all samples, Δ curves have a well-defined S-shape. Irrespective of the excitation wavelength, the higher the terbium content in the sample, the higher the Δ value at 83 K (Figure S7). To get qualitative information on the thermometric response among the mixed samples, the Δ curves were normalized at 83 K (Figure 8a,b).

In the mixed samples, the normalized Δ curves showed similar S-shapes regardless of the relative europium/terbium amount and excitation wavelength. As a result, the S_r_ vs. T curves, and hence the thermometric properties, of the different samples are quite similar (Figure 8c,d). All the S_r_ curves showed an asymmetric bell shape with a S_r_ maximum (S_rmax_) of 2.4% K^−1^ ca. between 303 and 323 K with an extended applicative temperature range (Sr ≥ 1, 100 K ÷ 165 K wide), depending on the sample composition (see Table 2).

Interestingly, excellent S_r_ values in the physiological temperature range (298–318 K) [27,47] were found for these ratiometric thermometers.

**Figure 9:**
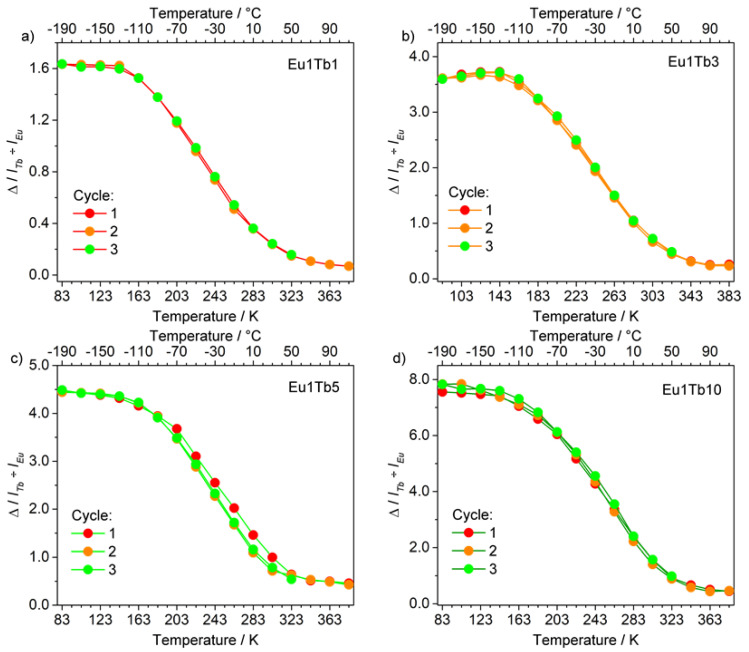
Heating/freezing cycles for samples (**a**) **Eu1Tb1**, (**b**) **Eu1Tb3**, (**c**) **Eu1Tb5**, and (**d**) **Eu1Tb10**. λ_exc_ = 320 nm.

The reproducibility of the thermometric response of the samples has been tested performing three different heating–cooling cycles on each sample. All the studied materials showed a highly reproducible thermometric response as well as the absence of thermal hysteresis (Figure 9).

The temperature resolution of our thermometers, i.e., the smallest detectable temperature variation (δT), was determined by the heating/freezing cycles, according to literature [2,3,18], using the following equation:

(2) $$\delta T=\frac{1}{S_{r}}\cdot \frac{\delta \Delta }{\Delta }$$

where δΔ is the Δ uncertainty at a given temperature [3,6] determined by calculating the standard deviation of three heating/freezing cycles. All the samples showed comparable resolution in the order of 0.01 K (average value over the thermometer operational range), in line with literature values for Ln-based luminescence thermometers [2,3].

**Figure 10:**
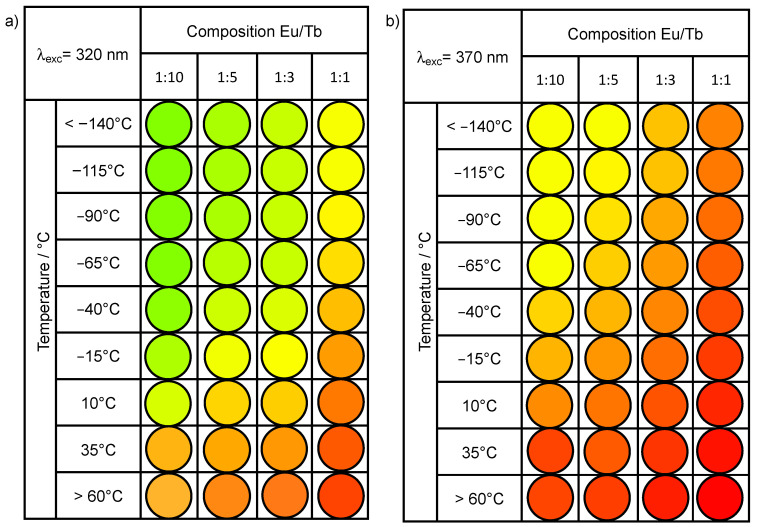
Overall chromatic variation for the studied samples excited at (**a**) 320 nm and (**b**) 370 nm.

The change of the excitation wavelength produces a variation of the samples color outputs. Indeed, at 370 nm, the green component due to terbium emission is less intense than at 320 nm, thus producing an overall chromatic shift towards red (Figure 10).

Figure 10 shows a schematic diagram representing the color variation of the different samples over the 83–383 K temperature range. The color at the different temperatures is defined by the CIE coordinates determined from the emission spectra obtained exciting the samples at 320 and 370 nm. Note how the temperature can be mapped by a chromatic reading generating something similar to the litmus paper, the famous pH testing papers, that can work with one or more chromatic channels simply defined by the composition of the mixture of **1** and **2**. Noteworthy, the correlation between the color of the emitted light and sample composition can also be used for the development of luminescent QR-codes [24] in which the temperature can be associated to a single color or a combination of two or more tints. In principle, the tuning of the europium/terbium relative amount should enable the optimization of the color gradient, thus allowing the detection of even narrower temperature intervals.

## 4. Conclusions

In this work, the temperature-dependent luminescent properties of the two homometallic 1D zigzag [Ln(hfac)_3_bipy]_n_ CPs (Ln^3+^ = Eu^3+^, 1, and Tb^3+^, 2) were studied in the 83–383 K temperature range. **1** and **2** showed *S_r_* values higher than 1 starting from 323 K and 223 K, respectively. The two homometallic compounds were then used to prepare a family of Eu^3+^/Tb^3+^ ratiometric thermometers in which terbium acts as probe and europium as reference. Four different thermometers were prepared by mixing different amounts of **1** and **2** in KBr matrix with Eu^3+^/Tb^3+^ molar ratio of 1:1, 1:3, 1:5, and 1:10, respectively. Sample composition and the excitation wavelength influenced the color of the emitted light but not the thermometric response. All the thermometers were sensitive to temperature variation between 83–383 K, showing a wide applicative range (S_r_ ≥ 1) from 100 K to 165 K depending on the sample composition. The samples showed high relative thermal sensitivity values in the physiological temperature range (298–318 K), with a S_r_ maximum value of about 2.40% K^−1^. The thermometric response of the samples did not vary significantly in changing the excitation wavelength that, instead, influences the color of the emitted light. The well-defined and peculiar color variation of heterobimetallic materials is used as a colorimetric thermometer. Using the eyes as a detector, temperature difference in the order of 10–20 K can be appreciated. To improve the temperature resolution, using the color of the emitted light as probe, calibrated luminance/color meters are required. The results here reported pose the basis for future activity oriented to bring luminescent molecular thermometer into daily life by measuring temperature by reading the color of the emitted light with a smartphone camera.

## Figures and Tables

**Table 1:** [Eu(hfac)_3_(bipy)]_n_ (**1**) and [Tb(hfac)_3_(bipy)]_n_ (**2**) amounts dispersed in KBr in the different samples.

Sample	[Eu(hfac)_3_(bipy)] (mmol)	[Tb(hfac)_3_(bipy)]_n_ (mmol)	KBr (mmol)
**1**	1.49 mg (1.60 × 10^−3^)	//	151.5 mg (1.27)
**2**	//	1.30 mg (1.39 × 10^−3^)	133.6 mg (1.12)
**Eu1Tb1**	1.05 mg (1.13 × 10^−3^)	1.04 mg (1.12 × 10^−3^)	209.6 mg (1.76)
**Eu1:Tb3**	0.55 mg (0.59 × 10^−3^)	1.65 mg (1.77 × 10^−3^)	222.8 mg (1.87)
**Eu1:Tb5**	0.59 mg (0.64 × 10^−3^)	2.99 mg (3.20 × 10^−3^)	362.1 mg (3.04)
**Eu1:Tb10**	0.28 mg (0.31 × 10^−3^)	2.90 mg (3.10 × 10^−3^)	325.3 mg (2.73)

**Table 2:** Applicative temperature range (S_r_ ≥ 1) and S_r_ maximum value (S_rmax_) of the studied Eu^3+^/Tb^3+^ mixed samples.

	λ_exc_ = 320 nm.		λ_exc_ = 370 nm.	
Sample	Applicative Temperature Range	*S_r_*_max_ (T)	Applicative Temperature Range	S_rmax_ (T)
**Eu1Tb1**	213–373 K	2.20 (303 K)	203–353 K	2.30 (303 K)
**Eu1Tb3**	223–363 K	2.10 (323 K)	243–343 K	1.95 (303 K)
**Eu1Tb5**	233–373 K	2.10 (303 K)	243–383 K	1.95 (323 K)
**Eu1Tb10**	233–373 K	2.40 (303 K)	233–363 K	2.60 (303 K)

## Data Availability

Not applicable.

## Supplementary Materials

The following are available online at https://www.mdpi.com/article/10.3390/ma14216445/s1, Figure S1: Superimposed infrared spectra of [Eu(hfac)_3_(bipy)]_n_ (**1**, red) and [Tb(hfac)_3_(bipy)]_n_ (**2**, green) CPs in the 2000–650 cm^−1^ wavenumber range; Figure S2: Excitation spectra of compound **1** (red) and **2** (green), λ_em_ = 614 and 543 nm, respectively. The vertical dashed lines highlight the two excitation wavelengths used in the work (320 and 370 nm); Figure S3: Room temperature emission spectra of samples (a) **Eu1Tb3**, (b) **Eu1Tb5** and (c) **Eu1Tb10**. λ_exc_ = 320 nm; Figure S4: Room temperature emission spectra of samples (a) **Eu1Tb3**, (b) **Eu1Tb5** and (c) **Eu1Tb10**. λ_exc_ = 370 nm; Figure S5: Temperature-dependent emission spectra of samples (a) **Eu1Tb3**, (b) **Eu1Tb5** and (c) **Eu1Tb10**. λ_exc_ = 320 nm; Figure S6: Temperature-dependent emission spectra of samples (a) **Eu1Tb3**, (b) **Eu1Tb5** and (c) **Eu1Tb10**. λ_exc_ = 370 nm; Figure S7: Δ vs. T curves of Eu-Tb mixed samples. (a) λ_exc_ = 320 nm, (b) λ_exc_ = 370 nm.
