# Supplementary material for: 1D-Zigzag Eu3+/Tb3+ Coordination Chains as Luminescent Ratiometric Thermometers Endowed with Multicolor Emission
Source: Materials (Basel). 2021 Oct 27;14(21):6445. doi: 10.3390/ma14216445 (PMC8585447; doi:10.3390/ma14216445)
Supplement: Supplementary file 1 [file materials-14-06445-s001.zip › materials-1425917-supplementary.pdf]

materials MDPI

Supplementary Materials

1D-Zigzag Eu^{3+}/Tb^{3+} Coordination Chains as Luminescent Ratiometric Thermometers Endowed with Multicolor Emission

Luca Bellucci [1,2,3], Gregorio Bottaro [1,3,*], Luca Labella [1,2,*], Fabio Marchetti [2], Simona Samaritani [2], Daniela Belli Dell'Amico [2] and Lidia Armelao [3,4]

[1] CNR ICMATE and INSTM, Dipartimento di Scienze Chimiche, Università di Padova, via Marzolo 1, I-35131 Padova, Italy; luca.bellucci@phd.unipd.it
[2] Dipartimento di Chimica e Chimica Industriale and CIRCC, Università di Pisa, via Giuseppe Moruzzi 13, I-56124 Pisa, Italy; fabio.marchetti@unipi.it (F.M.); simona.samaritani@unipi.it (S.S.); daniela.belli@unipi.it (D.B.D.)
[3] Dipartimento di Scienze Chimiche, Università di Padova, via Marzolo 1, I-35131 Padova, Italy; lidia.armelao@unipd.it
[4] Dipartimento di Scienze Chimiche e Tecnologie dei Materiali (DSCTM), Consiglio Nazionale delle Ricerche, Piazzale A. Moro 7, 00185 Roma, Italy
* Correspondence: gregorio.bottaro@cnr.it (G.B.); luca.labella@unipi.it (L.L.); Tel.: +39-049-8275275 (G.B.); +39-050-2219262 (L.L.)

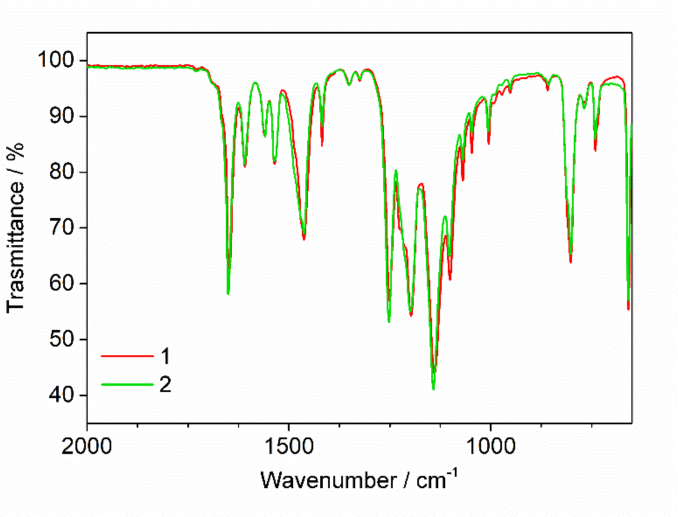

Figure S1. Superimposed infrared spectra of $[Eu(hfac)_3(bipy)]_n$ (1, red) and $[Tb(hfac)_3(bipy)]_n$ (2, green) CPs in the 2000–650 cm^{-1} wavenumber range.

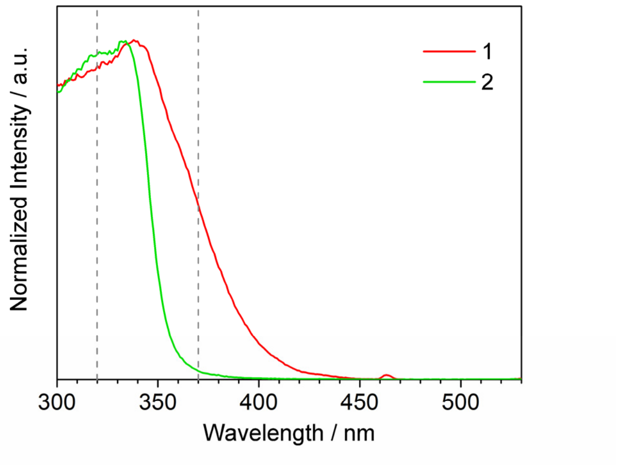

Figure S2. Excitation spectra of compound 1 (red) and 2 (green), λ_{em} = 614 and 543 nm, respectively. The vertical dashed lines highlight the two excitation wavelengths used in the work (320 and 370 nm).

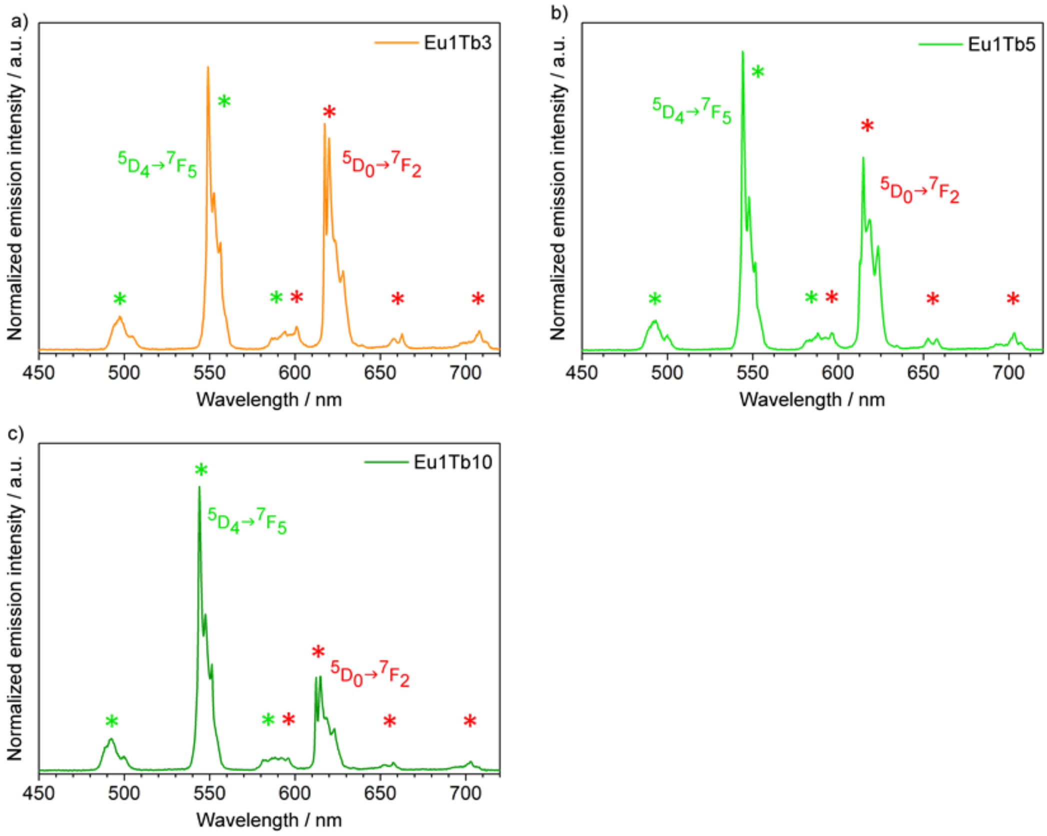

Figure S3. Room temperature emission spectra of samples (**a**) Eu1Tb3, (**b**) Eu1Tb5 and (**c**) Eu1Tb10. λ_{exc} = 320 nm.

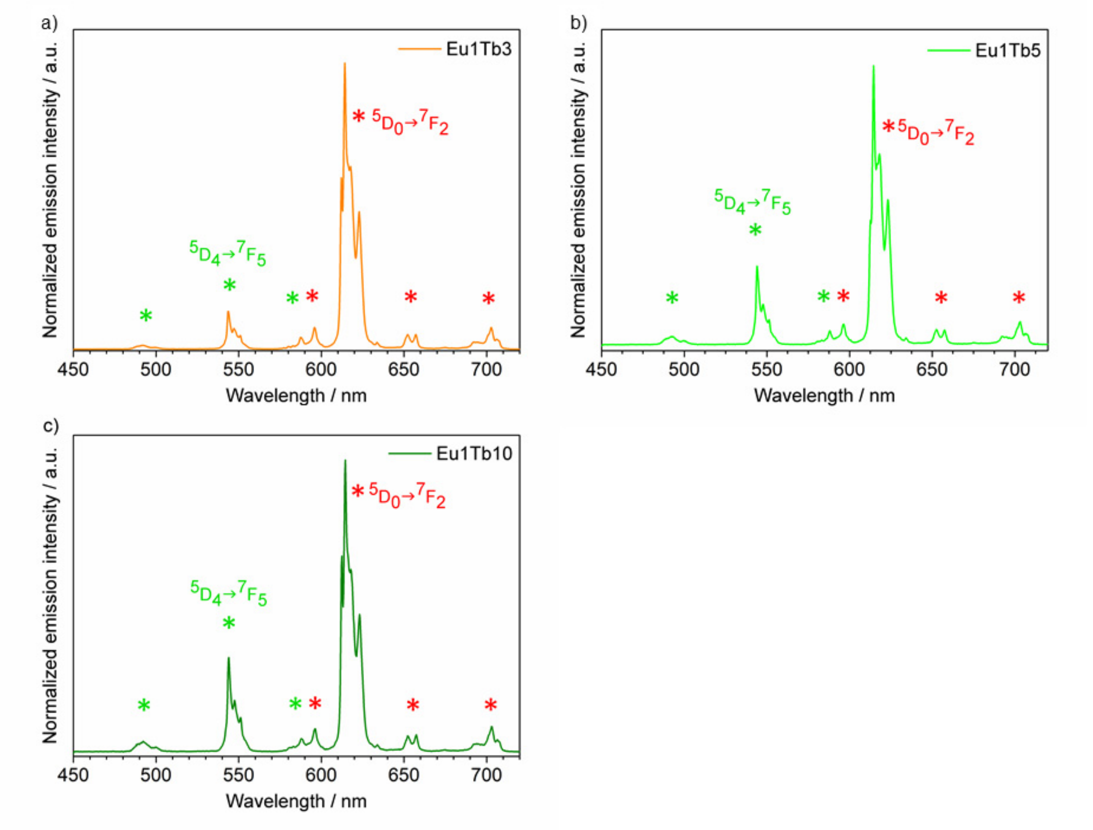

Figure S4. Room temperature emission spectra of samples (**a**) Eu1Tb3, (**b**) Eu1Tb5 and (**c**) Eu1Tb10. λ_{exc} = 370 nm.

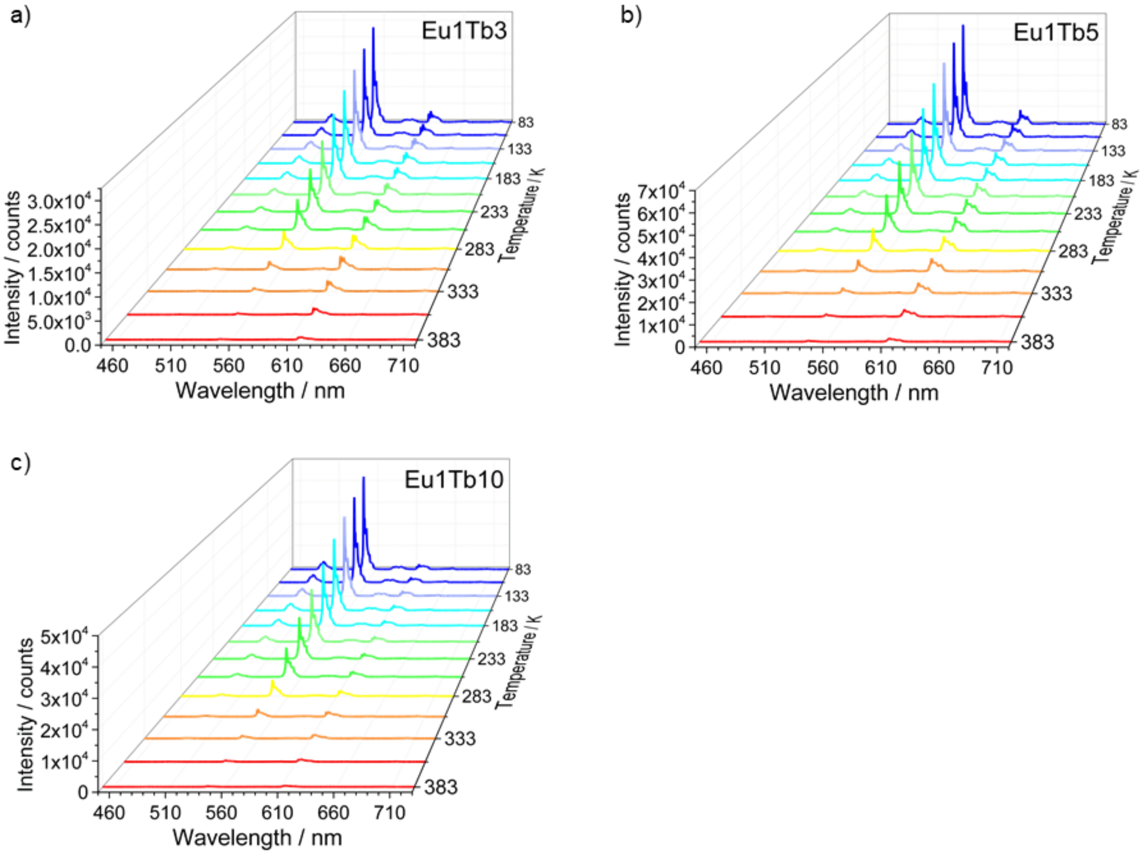

Figure S5. Temperature dependent emission spectra of sample (**a**) Eu1Tb3, (**b**) Eu1Tb5 and (**c**) Eu1Tb10. λ_{exc} = 320 nm.

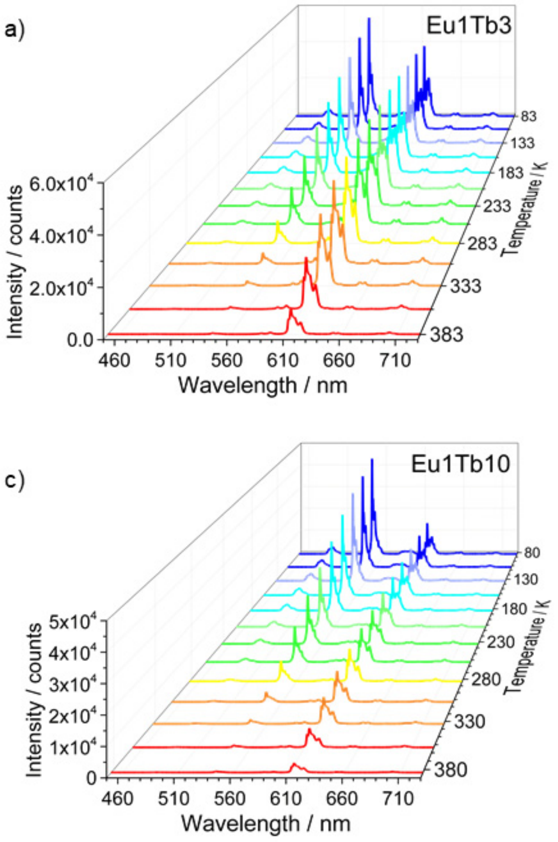

Figure S6. Temperature dependent emission spectra of sample (**a**) Eu1Tb3, (**b**) Eu1Tb5, (**c**) Eu1Tb10. λ_{exc} = 370 nm.

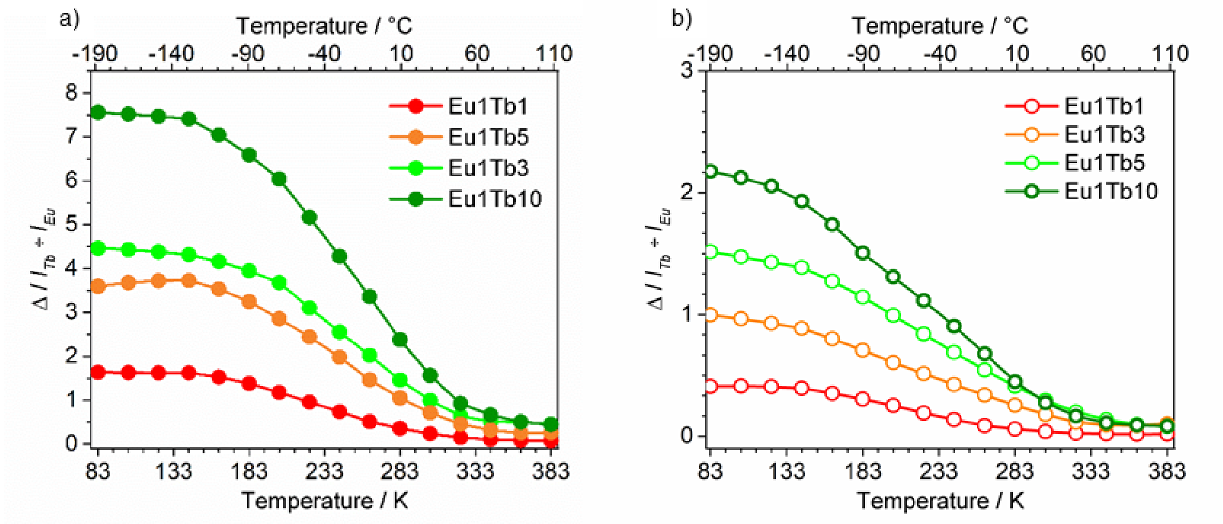

Figure S7. Δ vs T curves of Eu-Tb mixed samples. (**a**) λ_{exc}= 320 nm, (**b**) λ_{exc} = 370 nm.
